# Chitosan-Based Biocomposite Hydrogels with Squid Pen Protein for Anionic Dyes Adsorption

**DOI:** 10.1021/acsmaterialslett.4c01802

**Published:** 2025-02-14

**Authors:** Pedro Y. S. Nakasu, Maite A. Martinez, Susiana Melanie, Talia A. Shmool, Jason P. Hallett

**Affiliations:** Department of Chemical Engineering, Imperial College London, SW7 2AZ London, U.K.

## Abstract

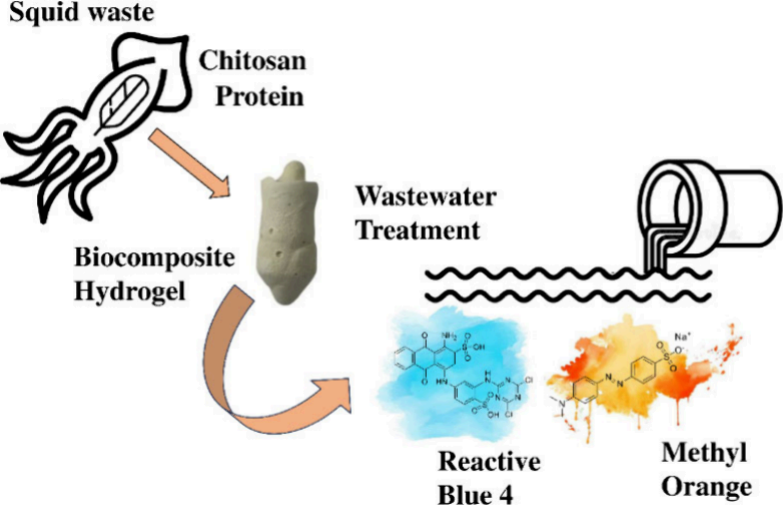

Growing environmental concerns have driven the search for sustainable wastewater treatment solutions, particularly for the removal of persistent synthetic dyes. This study explores hydrogels made from squid pen protein (SPP) and chitosan, biodegradable polymers, for anionic dye adsorption—reactive blue 4 (RB4) and methyl orange (MO). A 50%/50% SPP/chitosan hydrogel was optimal for RB4 adsorption while minimizing chitosan use. Adsorption followed the Langmuir model, with capacities of 151.52 mg/g for RB4 and 54.94 mg/g for MO. Optimal RB4 adsorption conditions were 65 °C, 6 h, pH 7, and 0.2 wt % adsorbent at 300 rpm. Kinetic analysis indicated a pseudo-second-order model, suggesting chemisorption. Characterization (Fourier Transform Infrared Spectroscopy - FT-IR, Scanning Electron Microscopy - SEM, X-ray Photoelectron Spectroscopy - XPS) revealed functional groups and binding mechanisms, with XPS confirming a nucleophilic attack from the between amino groups of chitosan/SPP protein and the dichlorotriazine moiety of RB4. Higher cross-linker content reduced adsorption. This study demonstrates SPP/chitosan hydrogels as a cost-effective and sustainable alternative for wastewater treatment.

In the wake of escalating environmental concerns, the quest for sustainable and eco-friendly solutions to water pollution has intensified.^1^ Among the myriad pollutants, synthetic dyes pose a significant challenge due to the persistence and adverse effects of these on the ecosystems and human health.^2^ Textile effluent dye concentrations are reported throughout a broad range of values. The textile effluent has a dye content of 10–50 mg/L (or 10–50 ppm). However, it has been claimed that 60 mg/L of reactive dyes is released from cotton factories.^3^ Such a concentration range of common dyes, including the anionic dyes reactive blue 4 (RB4) and methyl orange (MO) (structures shown in Figures S1 and S2 and Supporting Information (ESI)) is sufficient to cause toxicity issues to both the aquatic life and humans.^4,5^ Addressing this challenge necessitates innovative approaches that not only efficiently remove dyes from wastewater, but also adhere to principles of environmental sustainability.^6,7^

Hydrogels, three-dimensional networks capable of absorbing and retaining large volumes of water or aqueous solutions, have emerged as promising materials for wastewater treatment.^2,8^ Given the tunable properties of the hydrogels, these are attractive candidates for environmental remediation applications. Particularly, the synthesis of biocomposites offers a compelling avenue for designing hydrogels with enhanced functionality for dye removal due to the biocompatibility and biodegradability of hydrogels.^9^

In this study, we explore the synergistic potential of squid pen protein (SPP) and chitosan, two biocompatible and biodegradable polymers, in the fabrication of hydrogels tailored for the removal of anionic dyes from aqueous solutions. Squid pen protein (SPP), a promising stream extracted from squid pen, is readily available and an inexpensive marine waste composed of mainly 75 wt % protein and 25 wt % chitin.^10^ It offers a sustainable alternative to traditional synthetic polymers. When combined with chitosan, derived from the exoskeletons of crustaceans, the resulting hydrogels exhibit promising characteristics for dye sorption, owing to the complementary properties of the constituent polymers.^2^

The current state-of-the-art chitosan-based hydrogels for wastewater purification consists of chemically modifying chitosan by either adding additional amine functionalities, such as binding chitosan with 2-chloroethyl amine in the so-called polymer grafting, or impregnating chitosan hydrogels with cationic surfactants such as hexadecylamine.^11−15^ Both methods utilize chemicals derived from oil, which are not ideal from an environmental perspective.

The rationale behind this approach stems from the imperative to harness natural resources efficiently while mitigating the environmental footprint of wastewater treatment processes. Squid pen can be used to produce chitosan, and the protein is extracted from the pen with alkaline solutions that become wastewater, which negatively impact the process and the environment.^16^ By leveraging the unique amino acid functionalities of SPP and the structural integrity of chitosan-based materials, we aimed to develop hydrogels that not only demonstrate superior dye removal efficiency but also align with the principles of green chemistry and a circular economy. Additionally, chitosan cost can be as high as 200 USD per kg,^17^ and with up to 50 wt % of chitosan being substituted by an inexpensive squid waste and outperforming the pure chitosan hydrogels, we can upscale squid waste utilization in a more economically feasible manner.

The scope of this paper is to synthesize and characterize SPP-chitosan hydrogels and investigate the efficacy of these materials in removing the anionic dyes RB4 and MO from aqueous solutions. Through a systematic analysis of the physicochemical properties and dye sorption kinetics of the hydrogels, we elucidate the underlying mechanisms governing the dye removal process. Furthermore, we discuss the implications of our findings in the context of sustainable wastewater treatment strategies and outline potential avenues for future research.

We produced the biocomposite hydrogels by homogenizing both the SPP and chitosan in diluted acetic acid; then, a certain amount of the cross-linker glutaraldehyde was added. The final solution was then freeze-dried to yield a spongy foam (aerogel) that later was hydrated into a hydrogel for the dye adsorption experiments. The complete synthesis procedure of the hydrogels are described in the ESI.

We employed the design of experiments (DOE) to identify the optimal combination of component concentrations to produce hydrogels with superior dye adsorption capacity and physical attributes. For the synthesis of the SPP hydrogels, 11 different experiments were conducted consisting of 8 runs and 3 center point replicates; the standard error from the center point was employed as the standard error of the DOE points. The chitosan to protein percentage ratio, X1, ranged from 50 to 70 wt %, the cross-linking percentage content, X2, from 1 to 3 wt %, and the total solids loading, X3, from 1 to 5 wt %. The complete experimental matrix is shown in Table S1. The samples were named after the following DoE factors: X1_X2_X3, for instance, a sample with a chitosan to protein ratio, X1, of 70%, a cross-linker percentage, X2, of 2% and a total solid loading, X3, of 5% is named sample 70_2_5. To validate the contributions of the added proteins to enhance the adsorption capacity and physical strength, hydrogels with only chitosan were also prepared. The hydrogel formulation included a solid loading of 3% chitosan and 2% cross-linker, reflecting the center points from the DOE.

Once manufactured, the hydrogels underwent various tests to evaluate the effects of the selected variables and the hydrogel combinations on the characteristics and capacities of the hydrogels. These tests included mechanical strength, physical stability, water swelling ratio, and dye adsorption capacity. The complete characterization procedure of the hydrogels is described in the ESI.

## Water Absorption Capacity

The chitosan hydrogels (CH-1, CH-2, and CH-3) exhibited an average swelling of 110.7%. In contrast, the SPP hydrogels (60_2_3, 70_3_50, and 70_3_51) demonstrated an average swelling of 139.7 wt %. This increase in absorption capacity is attributed to the addition of each protein, which enhances its desirability as adsorbent of the hydrogels.

Moreover, it was observed that the solid loading significantly affects the swelling of the hydrogels. The SPP hydrogels with a 5% solid loading (70_3_5, 70_1_5, 50_3_5, and 50_1_5) and a 1% solid loading (70_3_1, 70_1_1, 50_3_1, and 50_1_1) exhibited average swelling of 140.7% and 71.7%, respectively. Overall, the protein-containing hydrogels with the lowest solid loading exhibited inferior results compared to even chitosan hydrogels, indicating that this composition does not yield valuable and efficient hydrogels in terms of absorption capacity.

Standard chitosan and acetic acid hydrogels have a water absorption capacity range of 90–600%, depending on the additional materials included in the formulation.^18^ In a study given in ref (19) with chitosan-*graft*-poly(acrylic acid), synthesized hydrogels had up to 160 wt % swelling^20^ and also water absorption capacities of up to 475 wt % for chitosan hydrogels with blue crab protein. Given this range, the values obtained for the SPP hydrogels were relatively low, possibly due to the presence of GLA in the formulation. Although GLA can provide increased stability, the dense network formed within the hydrogel can restrict the entry of water molecules, thereby reducing the water absorption capacities.

## Physical Integrity of Hydrogels

Both before and after the swelling stage, the physical stability of the hydrogels was evaluated. Hydrogels with a 5% solid loading (70_3_5, 70_1_5, 50_3_5, and 50_1_5), as well as the center point samples (60_2_3) and the pure chitosan hydrogels (CH-1, CH-2, and CH-3), demonstrated the most promising results in terms of mechanical integrity. During preparation, these gels formed a highly viscous, gel-like mixture upon the addition of both chitosan and cross-linker, indicating that the gelation point had been reached. This was attributed to the formation of a three-dimensional network structure within the hydrogel.

After lyophilization and with an average moisture content of 0.5 wt %, these gels exhibited a styrofoam-like consistency (Figure S3). The aerogels were extremely lightweight and rigid, retaining shape under minimal pressure. However, the aerogels displayed limited elasticity, resulting in permanent cracks and indentations when subjected to higher pressure.

Once hydrated, these hydrogels had a sponge-like consistency due to high porosity and visual texture (Figure S3), although the hydrogels were slightly dense. High porosity is a desirable characteristic for adsorbents due to the increased surface area. Nevertheless, the hydrogels were not soft and compressible and as such did not revert to their original shape when heavily compressed. Despite this, the three-dimensional network structure of these hydrogels showed promising results in terms of physical stability, potentially enabling the hydrogels to withstand adsorption capacity tests.

In contrast, hydrogels with a 1% solid loading (70_3_1, 70_1_1, 50_3_1, and 50_1_1) remained as slightly viscous liquids, even after the addition of chitosan and cross-linker, indicating that the gelation point was not reached. The incomplete formation of the three-dimensional network could be due to the low percentage of solids in the hydrogels. After lyophilization, these hydrogels exhibited significantly low porosity yet were lightweight, soft, and highly compressible. Once hydrated, these hydrogels barely maintained shape, and displaying slimy and mucilaginous properties. Such characteristics are undesirable for conducting adsorption tests due to the potential dissolution of the hydrogels when introduced into dye solutions.

Based on these findings regarding the physical stability and properties of the hydrogels, in conjunction with the water absorption capacity results, 70_3_1, 70_1_1, 50_3_1, and 50_1_1 were eliminated for investigation byfrom the dye adsorption efficiency tests.

## Dye Adsorption Capacity and Removal Efficiency

Following the first elimination stage, where water absorption capacity and physical stability of the hydrogels were considered, the dye adsorption capacity of the hydrogels was then evaluated with RB4 and MO solutions. These two anionic dyes were selected due to the structure (dichlorotriazine and azo dye) and utilization, serving as these relevant model molecules for dye adsorption.^21,22^ The results obtained during adsorption capacity and removal efficiency tests for the selected hydrogels and with a 100 ppm RB4 solution are presented in Table 1.

**Table 1:** Equilibrium Adsorption Capacity (*q*_e_) and Removal Efficiency (*R*) for Anionic Dyes RB4 and MO with Initial Concentration of 100 ppm, 0.2 wt % Hydrogel Loading for 24 h, and 300 rpm Stirring Speed

Dye	Sample	*q*_e_ (mg/g)	*R* (%)
	70_3_5	41	88
	70_1_5	46	98
	50_3_5	42	91
	50_1_5	46	99
RB4	60_2_3	40	87
	CH-1	39	85
	CH-2	39	84
	CH-3	40	85
	70_3_5	38	48
	70_1_5	55	70
	50_3_5	28	35
MO	50_1_5	44	56
	60_2_3	31	39
	CH-1	9	21
	CH-2	10	19
	CH-3	11	17

The addition of SPP resulted in superior adsorbance performance for both dyes, with samples 70_1_5 and 50_1_5 displaying nearly total removal efficiencies of 98% and 99% for RB4 and 70% and 56% for MO, respectively. As mentioned previously, these two hydrogels presented the lowest concentration of cross-linker (1 wt %) during synthesis. Therefore, the availability of nucleophilic sites in both SPP and chitosan is higher to bind to the dyes. *q*_e_ values also correlated to the removal efficiencies with maximum values of 46 mg of RB4/g of hydrogel and 55 mg of MO/g of hydrogel 70_1_5.

The SPP-chitosan hydrogels were more interactive toward RB4 compared to MO; this could be explained by the fact that reactive dyes present moieties can potentially covalently bind to the adsorbent active sites. For instance, the dichlorotriazine moiety can be attacked by nucleophilic groups in the biocomposite hydrogels.^23,24^ MO, on the other hand, shares only the aromatic moieties and the sulfonate group for dye-adsorbent interactions.

Considering the adsorption capacity and removal efficiency results for both RB4 and MO solutions and the zeta potential values of the adsorbent components and dyes (in depth discussion in section 3.1 in the ESI), both dyes were chosen as the probe dyes for the adsorption isotherms. Additionally, hydrogels 70_1_5 and 50_1_5 were selected for adsorption isotherm analysis due to the superior performance of these formulations.

## Adsorption Isotherms

**Figure 1:**
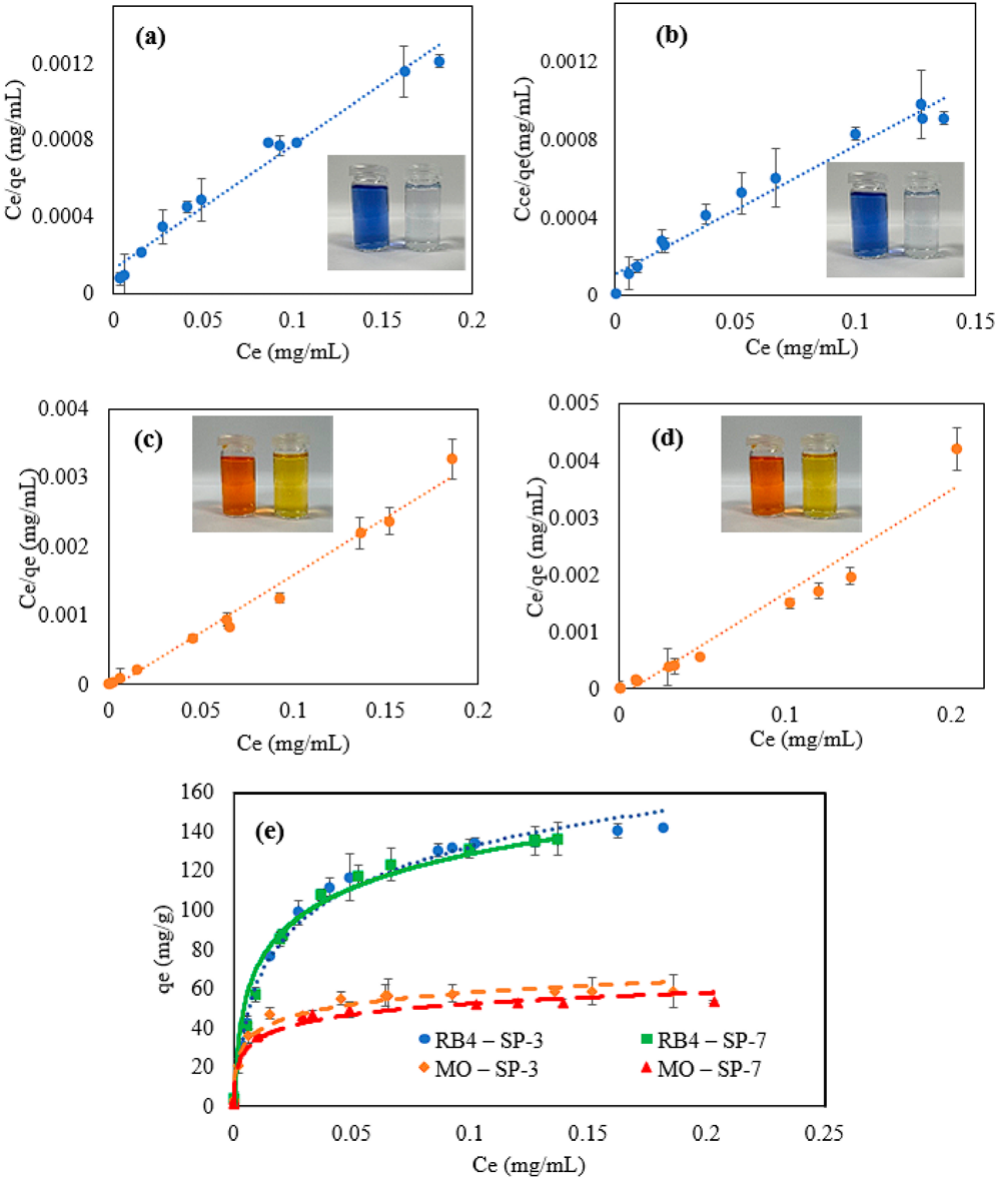
Equilibrium adsorption isotherms performed with 0.2 wt % hydrogel loading for 24 h and 300 rpm stirring speed. (a) RB4 adsorption by hydrogel 70_1_5. (b) RB4 adsorption by hydrogel 50_1_5. (c) MO adsorption by hydrogel 70_1_5. (d) MO adsorption by 50_1_5. (e) Langmuir isotherms.

To characterize the adsorption behavior of samples 70_1_5 and 50_1_5 with both RB4 and MO solutions, respectively, adsorption isotherms based on Langmuir theory were determined and analyzed. By plotting the experimentally obtained data of *C*_e_ and *q*_e_ according to the Langmuir model, Figures 1a–d was generated. Using the data from these plots and eq 6 in the ESI, the Langmuir constants *K*_L_ and α_L_ were calculated. The linearity of each plot was evaluated based on the *F* and *R*^2^ values of each trendline, which were 410, 308, 783, and 190 for the *F* values and 0.97382, 0.9654, 0.9863, and 0.9449 for the *R*^2^ values in Figure 1a–d, respectively.

**Figure 2:**
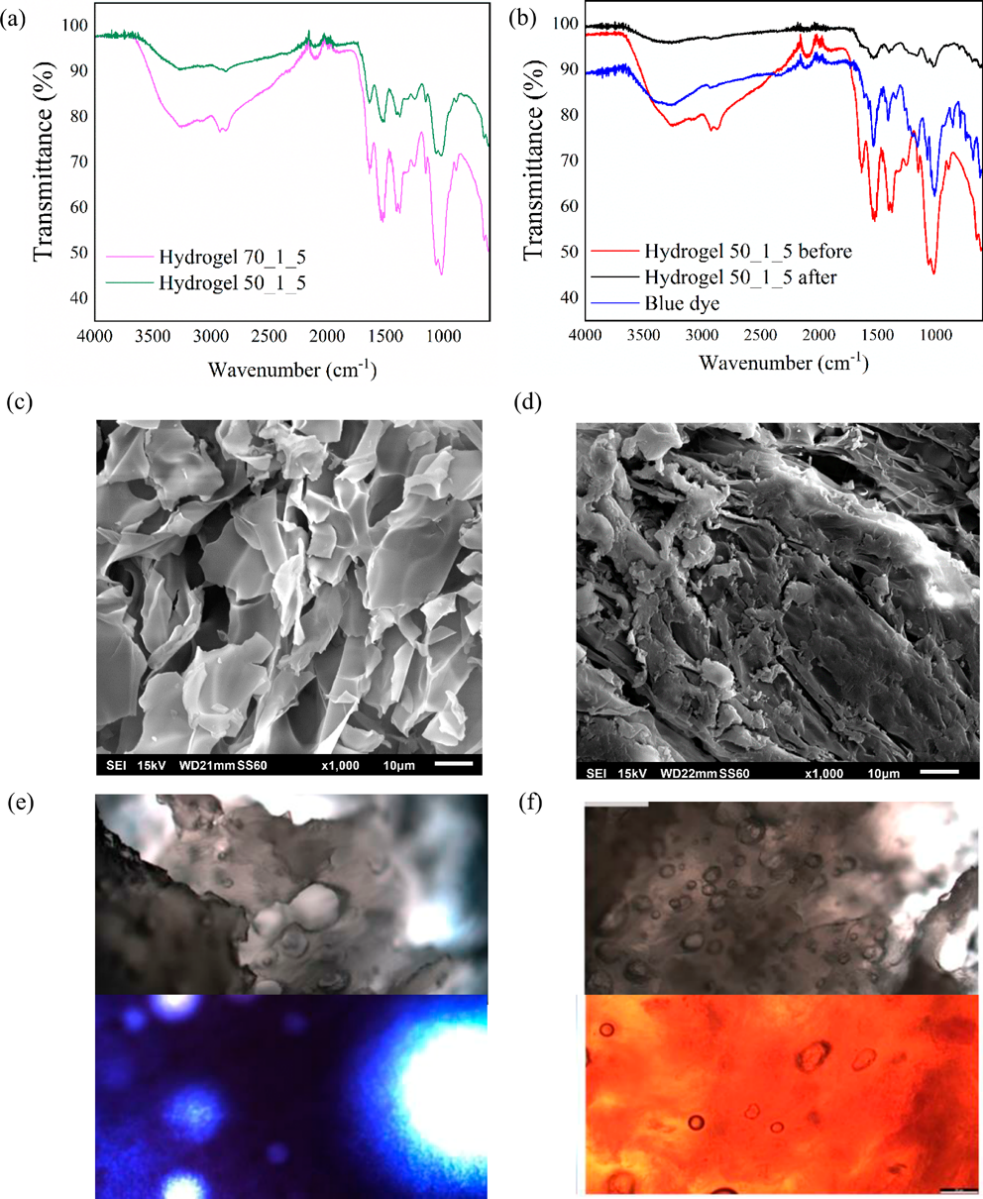
Characterization of the hydrogels produced with an initial dye concentration of 100 ppm, 0.2 wt % hydrogel loading for 24 h, and 300 rpm stirring speed. (a) FT-IR spectra of the dry hydrogels. (b) RB4 adsorption by hydrogel 50_1_5 (before and after). (c) SEM of chitosan hydrogel with ×1000 magnification. (d) SEM of hydrogel 50_1_5 with ×1000 magnification. (e) Optical image of hydrogel 50_1_5 before and after RB4 adsorption. (f) Optical image of hydrogel 50_1_5 before and after MO adsorption.

Through the linearization of the experimental data, it was possible to plot the adsorption isotherms, following Langmuir theory, for both 70_1_5 and 50_1_5, with RB4 and MO solutions, as illustrated in Figure 1e. The substantial adsorption capacity of both samples is evident in Figure 2, characterized by the sharp and steep initial increase, followed by complete monolayer formation indicated by the plateau in each plot. In a work by Galan et al.^25^ on the adsorption of RB4 with chitosan beads cross-linked with glutaraldehyde, a Freundlich isotherm was deemed as the optimal linear fit for the adsorption process. This model posits that instead of a monolayer formation, there is a heterogeneous distribution of the active sites on the surfaces of the chitosan beads. Additionally, in a study by Zhai et al. on the adsorption of MO with chitosan microspheres,^11^ the Langmuir model better described sample adsorption process, which indicates behavior similar to that in this work.

The calculated *q*_max_ values for experiments RB4-70_1_5, RB4-50_1_5, MO-70_1_5, and MO-50_1_5 were 153.85, 151.52, 60.24, and 54.94 mg/g, respectively. These *q*_max_ values for RB4 were superior to those in studies that also entailed producing bioadsorbents such as pure chitosan beads, *q*_max_ 1.56 mg/g, chitosan and hydroxyapatite biocomposites, *q*_max_ 118.4, and 100% deacetylated chitosan, *q*_max_ 54.01 (Table 3). However, these were still inferior to those in studies that derivatized chitosan such as the works given in refs (12, 13, and 26). Regarding MO, similar trends were observed with an underivatized chitosan hydrogel, 12.46 mg/g.^23^ Whereas with modified chitosan biomaterials, results varied from a lower *q*_max_ of 10.53^27^ to higher values of 900 mg/g such as the one produced by Chen et al.^28^ with a cerium-based metal organic framework chitosan polyimine biocomposite.

## Effect of Different Parameters on the Adsorption of RB4 to Hydrogel 50_1_5

### Effect of Temperature

Hydrogel 50_1_5 was chosen due to its superior performance in conjunction with hydrogel 70_1_5 on RB4 adsorption. However, a lower chitosan content ends up lowering the cost of the hydrogel (50% chitosan instead of 70%). The effect of temperature is shown in Figure S5. An increase in 20 °C accelerates the adsorption of RB4, as it can be seen that at 45 and 60 °C, the RB4 dye concentration reaches a minimum at 12 and 6 h. An endothermic adsorption provides a hint about the type of adsorption, which is most likely chemisorption. The kinetics data were then analyzed for adsorption at 65 °C.

### Kinetics of RB4 Adsorption

The kinetics of the RB4 adsorption have been investigated in terms of fitting to the pseudo first-order or second-order models. The fitting parameters are shown in Table 2.

**Table 2:** Kinetic Model Parameters for RB4 Adsorption

Pseudo-First-Order			
*q*_e_ (mg/g)	*k*_1_ (L/min)	*R*^2^	adj *R*^2^
117.77	3.352	0.62	0.55

Pseudo-Second-Order			
*q*_e_ (mg/g)	*k*_2_ (mg/(min min))	*R*^2^	adj *R*^2^
133.54	0.00534	0.88	0.85

It can be noticed that the pseudo-second-order model presented a better fit with an adjusted *R*^2^ of 0.85 compared to 0.55 of the pseudo-first-order model. In Figure S6, the fitting of the models can be visualized, showing the predicted curves compared with the experimental data. The pseudo-second-order model considers chemisorption as the rate-limiting mechanism of the process.

### Effect of pH

There is a close relationship between the pH and the charge of the particles in aqueous solutions. The effect of pH can be seen in Figure S7. For pH values below 4 (1 and 2), the adsorption was inefficient. This can be explained by the net charge of RB4 at low pHs, where the amino groups (there are four in total) become fully protonated and the dye is no longer anionic; therefore, there is electrostatic repulsion between the hydrogel and RB4.

### Effect of Adsorbate Dose

The effect of initial concentration of RB4 can be seen in Figure S8. Up to 100 ppm of the dye can be fully adsorbed onto the hydrogel; however, at 150 ppm, the efficiency considerably drops, which may hint at the saturation of the hydrogel surface.

### Desorption: Regeneration of the Hydrogel

Regeneration of the adsorbent is important to prove the reusability of the materials. Attempts have been made with several organic solvents such as ethanol, acetone, and diethyl ether. Other less conventional solvents such as ionic liquids (choline acetate) have also been proven; however, RB4 did not desorb from the materials, proving the chemisorption was indeed the mechanism of interaction.

## Hydrogel Characterization

The samples 70_1_5 and 50_1_5 chemical structure and surfaces were then characterized through Fourier transform infrared (FT-IR) spectroscopy, optical microscopy, scanning electron microscopy (SEM) imaging, and XPS analysis.

### FT-IR Spectroscopy

The FTIR spectra of dry hydrogels 70_1_5 and 50_1_5, illustrated in Figure 2a, show that both samples have the same absorption bands due to the same composition. The most significant absorption bands were found at 3251, 2866, 2102, 1635, 1521, 1399, 1375, 1248, 1151, 1062, 1017, and 889 cm^–1^, which are further characterized in Table S11. Specifically, the amide I C=O stretching at 1635 cm^–1^, the amide II N–H bending at 1511 cm^–1^, and the C–O–C and C–O stretching between 950 and 1200 cm^–1^ were also present, which are functional groups highly abundant in proteins, as also found in previous work.^29^ Even though similar, it can be noticed that sample 70_1_5 presents higher intensity on the bands at 3251, 1635, and 1521 cm^–1^ which correspond to the higher content of chitosan. After RB4 dye adsorption, the spectra of dry 70_1_5 and 50_1_5 hydrogels were compared to the original spectra, as well as the RB4 powder spectra; it was hard to spot the absorption bands of the dye due to the poor absorbance of the material.

### Optical Microscopy and Scanning Electron Microscopy (SEM)

SEM images of chitosan and hydrogel 50_1_5 are shown in Figure 2c,d. The surface of the chitosan hydrogel appeared rough and was composed of several chitosan microparticles. On the other hand, the surface of the 50_1_5 hydrogel, although rough, seemed to be more homogeneously distributed, and the microparticles could not be distinguished. Images of samples 70_1_5 and 50_1_5 were taken before and after dye adsorption with two different objective lenses to analyze the surfaces at various levels of detail. The presence of pores, ridges, and surface texture are crucial characteristics of an adsorbent, since these provide a larger surface area. This indicates that the hydrogel contains more binding sites to which the dye molecules adhere, resulting in superior adsorption capacity. Previously, the roughness of biopolymer-based hydrogels was also observed with chitosan and gelatin hydrogels.^27,28^ Optical microscopy images in Figure 2e,f show the presence of bubbles entrapped in the hydrogel matrix, which can help to increase hydrogel preferential site adsorption.

### XPS Analysis and Reaction Mechanism of Adsorption

**Figure 3:**
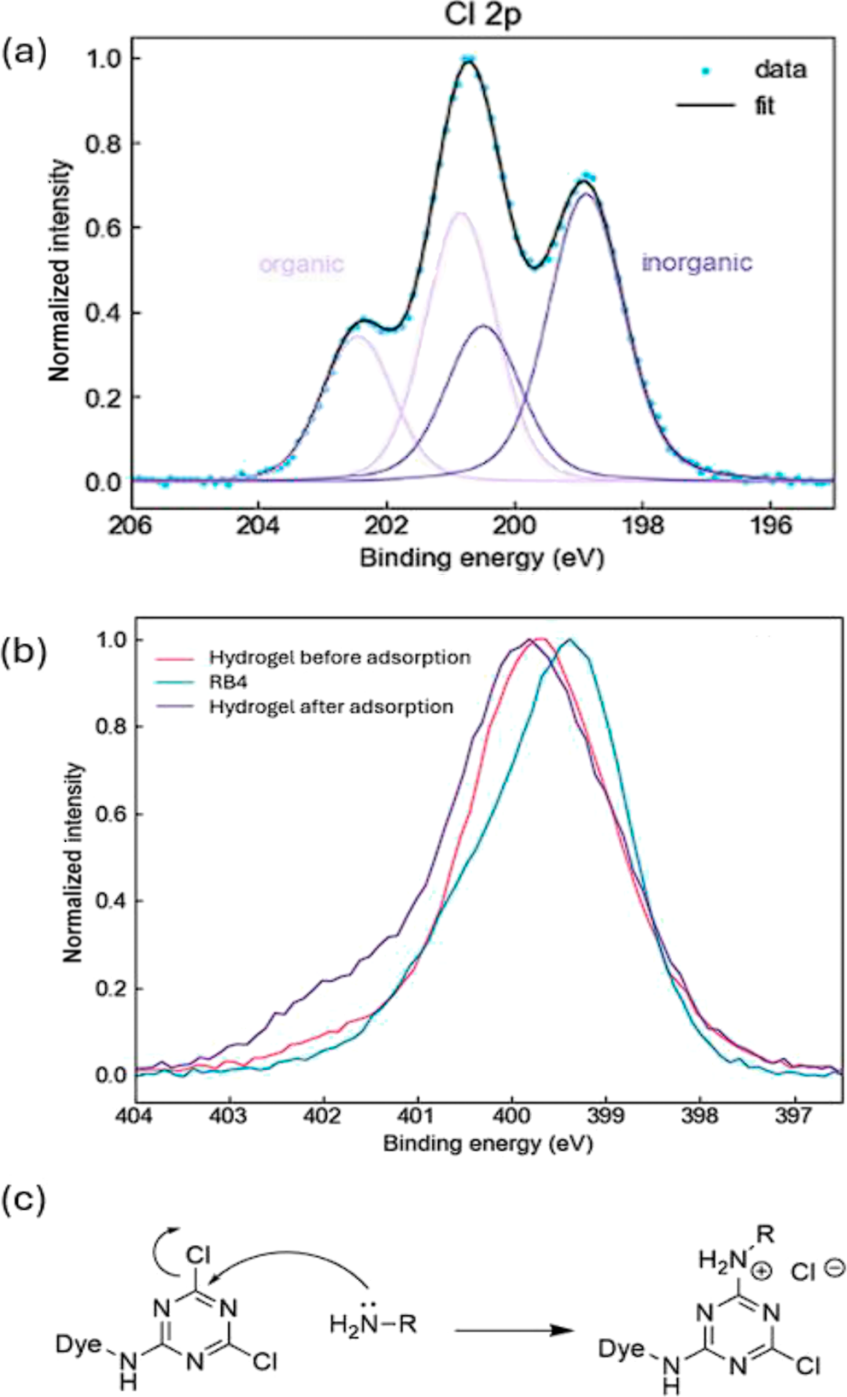
High-resolution XPS scans showing (a) Cl 2p from the hydrogel after adsorption and (b) N 1s from RB4 and the hydrogel before and after adsorption. (c) Reaction mechanism of the dye chemosorption. The R group may be either part of an amino acid residue or from chitosan.

XPS analysis is an excellent spectroscopic technique to unveil the surface chemistry of materials. The elemental composition from the low-resolution XPS survey is shown in Table S11. It can be seen that the chlorine content in RB4 is about 4.5%, which is slightly lower than the theoretical 11.1% due to the presence of impurities in the dye. However, the most striking difference is the increase of elemental chlorine in the hydrogel after adsorption, which indicates that chlorine from the dye has been incorporated in the hydrogel. There was also an increase in the nitrogen content, which also confirms the presence of the dichlorotriazine (at least with one chlorine atom) structure. The presence of the dye can also be confirmed by analyzing the high resolution spectra in Figure 3. From Figure 3a, it can be seen that chlorine is present in both organic and inorganic forms in the hydrogel after adsorption. This indicates that chloride anions could have been generated during the adsorption process via a nucleophilic attack of amino groups from either the chitosan or an amino acid residue, such as lysine to the dichlorotriazine moiety (Figure 3c). This leads to the final evidence, which is the increase in higher oxidation state nitrogen atoms (Figure 3b) in the hydrogel after adsorption, showing that cationic nitrogen (from the nucleophilic attack) may be present. The proposed mechanism is aligned with the currently accepted mechanisms for the reaction of RB4 with cellulose.^30^

## Industrial Feasibility of the Hydrogels

The biocomposite hydrogels presented promising results in terms of RB4 removal, especially at higher temperatures (65 °C), with complete removal of the dye in about 6 h. Scaling up of the hydrogel production would entail greater amounts of the SPP, which, at the moment, is limited due to squid supply being seasonal. However, there are other types of seafood waste that could have protein extracted such as fish and crustaceans such as shrimp, lobsters, and crabs. Alternative proteins could be harvested from seafood waste to produce more biocomposite hydrogels. A potential issue could be hydrogel regeneration, which has been proven irreversible. However, there are alternative end uses for these colored materials such as being processed into pigments for art supplies, coatings, or eco-friendly dyes for printing applications. or being used to fabricate composite materials for aesthetic applications in construction (e.g., colored concrete or tiles) or functional purposes (e.g., smart coatings).

We produced biobased composites comprised of chitosan and squid pen protein. The functionality from the amino acid residues enhanced the extraction of two anionic dyes, RB4 and MO, with dye removal efficiencies of up to 99% for RB4. Higher cross-linker content negatively affected the adsorption performance due to the loss of potential binding nucleophilic sites from both the protein and chitosan. Parametric studies show that the hydrogel reacts with RB4 via chemosorption, and the reaction is irreversible. However,

The combination of an alternative and cheap protein with chitosan into hydrogels paves the path for a more economically feasible wastewater treatment option.

**Table 3:** Comparison of Different Chitosan-Based Adsorbents for the Removal of RB4 and MO

Type of hydrogel	Dye	Adsorbent concentration (g/L)	Adsorbate concentration (mg/L)	pH	Maximum adsorption capacity (mg/g)	ref
Pure chitosan beads	RB4	0.4/0.025	55	3	1.56	(Galan et al. 2021)
Chitosan and hydroxyapatite	RB4	0.03	950	4	118.4	(Rastgordani and Zolgharnein 2021)
100% deacetylated chitosan	RB4	0.05/0.025	63.74	4	54.01	(Subarna Karmaker et al. 2020)
Chitosan/hexadecylamine	RB4	0.2/0.2	500	4	454	(Vakili et al. 2016)
This study	RB4	0.04/0.020	100	7	153.85	Nakasu et al.
Amino-ethyl chitosan	MO	0.3/0.015	100	2	5	(Tamer et al. 2024)
Polyethylenimine-modified chitosan	MO	0.020/0.020	1000	7	900.5	(Chen et al. 2023)
Magnetic chitosan-Fe_3_O_4_	MO	0.005/0.020	100	8	758	(Yang et al. 2016)
This study	MO	0.04/0.020	100	7	60.24	Nakasu et al.

## Supporting Information Available

Materials and methods, water absorption capacity of hydrogels, and hydrogel characterization (PDF)

## Abbreviations

GLA: Glutaraldehyde
MO: Methyl orange
SPP: Squid pen protein
RB4: Reactive blue 4
