# Correction to “Chitosan-Based Biocomposite Hydrogels with Squid Pen Protein for Anionic Dyes Adsorption”

**DOI:** 10.1021/acsmaterialslett.5c00937

**Published:** 2025-08-30

**Authors:** Pedro Y. S. Nakasu, Maite A. Martinez, Susiana Melanie, Talia A. Shmool, Jason P. Hallett

## Abstract

Growing environmental concerns have driven the search for sustainable wastewater treatment solutions, particularly for removing persistent synthetic dyes. This study explores hydrogels made from squid pen protein (SPP) and chitosan, biodegradable polymers, for anionic dye adsorption—reactive blue 4 (RB4) and methyl orange (MO). A 50%/50% SPP/chitosan hydrogel was optimal for RB4 adsorption while minimizing chitosan use. Adsorption followed the Langmuir model, with capacities of 151.52 mg/g for RB4 and 54.94 mg/g for MO. Optimal RB4 adsorption conditions were 65 °C, 6 h, pH 7, and 0.2 wt % adsorbent at 300 rpm. Kinetic analysis indicated a pseudo-second-order model, suggesting chemisorption. Characterization (FT-IR, SEM, XPS) revealed functional groups and binding mechanisms, with XPS confirming a nucleophilic attack between the amino groups of chitosan/SPP protein and RB4’s dichlorotriazine moiety. Higher cross-linker content reduced adsorption. This study demonstrates SPP/chitosan hydrogels as a cost-effective, sustainable alternative for wastewater treatment.

A typographical error has been corrected in the seventh sentence of the Abstract. The complete, corrected Abstract is provided here.

The Acknowledgment is also corrected to include an additional source of funding. The complete, corrected Acknowledgment is provided here.

