# Supplementary material for: Chitosan-Based Biocomposite Hydrogels with Squid Pen Protein for Anionic Dyes Adsorption
Source: ACS Mater Lett. 2025 Feb 14;7(3):1012–8. doi: 10.1021/acsmaterialslett.4c01802 (PMC11881141; doi:10.1021/acsmaterialslett.4c01802)
Supplement: Supplementary file 1 — tz4c01802_si_001.pdf [file tz4c01802_si_001.pdf]

ELECTRONIC SUPPORTING INFORMATION
CHITOSAN-BASED BIOCOMPOSITE HYDROGELS WITH SQUID PEN PROTEIN FOR ANIONIC DYES ADSORPTION

Pedro Y. S. Nakasu‡[1], Maite A. Martinez‡[1], Susiana Melanie[1], Talia A. Shmool1, Jason P. Hallett[1]

[1]Department of Chemical Engineering, Imperial College London, SW7 2AZ, London
‡These authors contributed equally

1.1 Dye structures

1.1.1 Reactive Blue 4 (1-Amino-4-[3-(4,6-dichlorotriazin-2-ylamino)-4-sulfophenylamino]anthraquinone-2-sulfonic acid)

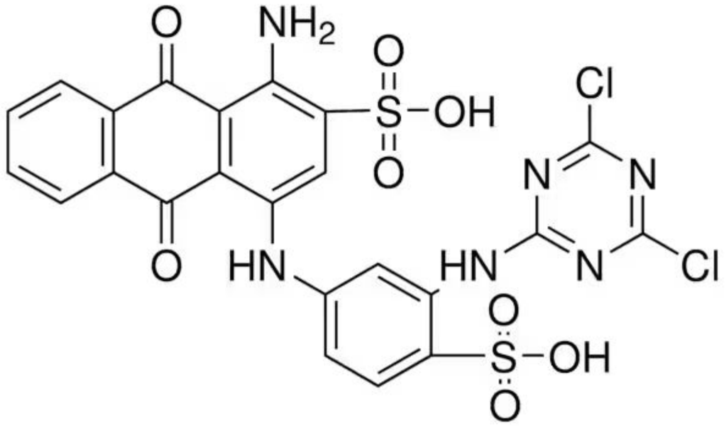

Fig. S1. Reactive Blue 4

1.1.2 Methyl orange (4-[4-(Dimethylamino)phenylazo]benzenesulfonic acid sodium salt)

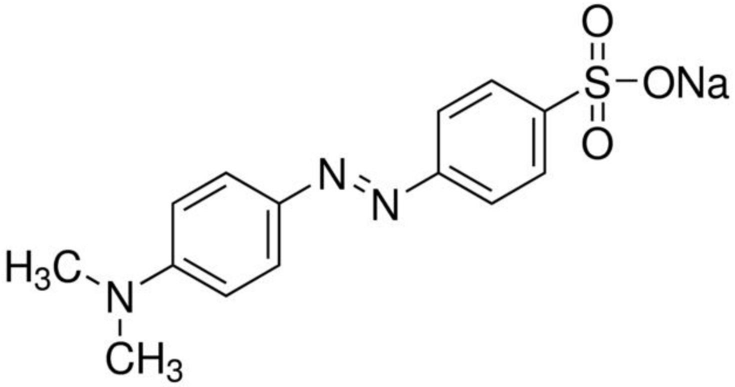

Fig. S2. Methyl Orange

1.2. Design of experiment

The design of experiment (DoE) approach was undertaken to find the optimum combination of all the component concentrations, that would result in the best hydrogel possible in terms of dye adsorption capacity and physical attributes[1]. First, the key independent variables involved in the gel composition and formulation were selected: chitosan to protein percentage ratio (X1), crosslinker percentage content (X2), and total solids loading (X3) – chitosan plus protein. For each variable, three reasonable values were selected, High (+), Center Point (0), and Low (-), as outlined in Table S1.

Table S1. Component parameters for each variable

Variable	+ (wt%)	0 (wt%)	- (wt%)
**Chitosan to protein percentage ratio (X1)**	70	60	50
**Crosslinker percentage content (X2)**	3	2	1
**Total solids loading (X3)**	5	3	1

Standard chitosan hydrogels were produced by solubilising 3 wt% of chitosan, being the total solid loading, in a 2 wt% acetic acid solution. From the conventional 3 wt%, a range of values was selected, both higher and lower, to explore a broader set of conditions whilst also minimizing the number of experiments(Fan et al. 2013). The GLA loading range was inspired by established studies, as well as by considering the inhibition of adsorption sites caused by GLA when added in large amounts. Therefore, the GLA 1% solution was added in small amounts of 1, 2 and 3 wt%(Gao et al. 2022). The total number of experiments, 11, was found through Equation (1).

$$(High + Low)^{Variables} + Center\ Points \qquad (1)$$
$$= 2^3 + 3$$
$$= 11\ experiments$$

Lastly, the formulation for each experiment is presented in Table S2. This DoE aims to explore every possible parameter combination, encompassing both high and low values for each variable. Furthermore, the outcome of the combination of the three centre points was tested and iterated thrice to ensure consistency, while creating an optimal region for the process(Allen et al. 2004).

Table S2. Hydrogels' formulation

Sample name	Chitosan to protein percentage ratio (X1)	Crosslinker percentage content (X2)	Total solids loading (X3)
70_3_5	70	3	5
70_3_1	70	3	1
70_1_5	70	1	5

70_1_1	70	1	1
50_3_5	50	3	5
50_3_1	50	3	1
50_1_5	50	1	5
50_1_1	50	1	1
60_2_3	60	2	3
60_2_3	60	2	3
60_2_3	60	2	3

To validate that the added proteins provide further adsorption capacity and physical strength, chitosan hydrogel controls without protein were also prepared. Their formulation included a 3% solids loading, of pure alpha chitosan, and a cross-linker percentage of 2% – the centre points from the DoE and the same parameters as samples 9, 10 and 11. The just chitosan hydrogels were produced thrice and named CH-9, CH-10 and CH-11. Once manufactured, the hydrogels underwent various tests to study the effect of the selected variables, with their different combinations, on the gels' characteristics and capacities. These included physical stability, water swelling ratio and dye adsorption capacity(Smith 2018).

1.3 Linear regressions – Adsorption isotherms

1.3.1 RB4 adsorption with hydrogel 70_1_5

Table S3. Summary statistics

	**Intercept**		**Slope**		**Statistics**
	**Value**	**Standard Error**	**Value**	**Standard Error**	**Adj. R-Square**
C_e/q_e	1.2455E-4	2.787E-5	0.00647	3.19598E-4	0.97383

Table S4. ANOVA of the linear regression

		**DF**	**Sum of Squares**	**Mean Square**	**F Value**	**Prob>F**
C_e/q_e	**Model**	1	1.74123E-6	1.74123E-6	410.33913	<0.0001
	**Error**	10	4.24339E-8	4.24339E-9		
	**Total**	11	1.78366E-6			

1.3.2 RB4 adsorption with hydrogel 5_1_5

Table S5. Summary statistics

	**Intercept**		**Slope**		**Statistics**
	**Value**	**Standard Error**	**Value**	**Standard Error**	**Adj. R-Square**

C_e/q_e	1.14302E-4	2.88037E-5	0.00658	3.75E-4	0.96537

Table S6. ANOVA of the linear regression

		DF	Sum of Squares	Mean Square	F Value	Prob>F
C_e/q_e	**Model**	1	1.27565E-6	1.27565E-6	307.59821	<0.0001
	**Error**	10	4.14713E-8	4.14713E-9		
	**Total**	11	1.31712E-6			

1.3.3 MO adsorption with hydrogel 70_1_5

Table S7. Summary statistics

	Intercept		Slope		Statistics
	**Value**	**Standard Error**	**Value**	**Standard Error**	**Adj. R-Square**
**Ce/qe**	-6.60632E-5	5.25558E-5	0.01659	5.89019E-4	0.9863

Table S8. ANOVA of the linear regression

		DF	Sum of Squares	Mean Square	F Value	Prob>F
**Ce/qe**	**Model**	1	1.28257E-5	1.28257E-5	792.86041	<0.0001
	**Error**	10	1.61764E-7	1.61764E-8		
	**Total**	11	1.29874E-5			

1.3.4 MO adsorption with hydrogel 50_1_5

Table S9. Summary statistics

	Intercept		Slope		Statistics
	**Value**	**Standard Error**	**Value**	**Standard Error**	**Adj. R-Square**
**Ce/qe**	-1.34579E-4	1.14375E-4	0.01816	0.00132	0.94492

Table S10. ANOVA of the linear regression

		DF	Sum of Squares	Mean Square	F Value	Prob>F
**Ce/qe**	**Model**	1	1.63792E-5	1.63792E-5	189.71902	<0.0001
	**Error**	10	8.63341E-7	8.63341E-8		

	**Total**	11	1.72426E-5			

2. Materials and Methods

2.1. Materials

Chitosan (75% deacetylated, alpha – from shrimp shells), glutaraldehyde solution (50% purity), reactive blue 4 (1-Amino-4-[3-(4,6-dichlorotriazin-2-ylamino)-4-sulfophenylamino]anthraquinone-2-sulfonic acid) (35% purity), methyl orange (85% purity) and hydrochloric acid (36% purity), Choline bicarbonate (98% purity) were all purchased from Sigma Aldrich (Merck KGaA, Darmstadt, Germany). Glacial acetic acid, ethanol, acetone and sodium hydroxide (98.8% purity) were purchased from VWR International (Leicestershire, England) and stored as recommended. Choline acetate was synthesized in a protocol similar to Nakasu et al. (2020) (Nakasu et al. 2020).

2.2. Squid pen protein extraction

The dry weight of the grounded squid pen was calculated and applied to a ratio of 5% of the squid pen to 95% of 0.5M NaOH solution. These were mixed for 3 hours, at 400 RPM and at room temperature. After the NaOH solution deproteinized the squid pen, the mixture was to filtered to separate the protein from the chitin and any existing minerals. The solid remainder, containing chitin and minerals, was washed with DI water until it reached a pH of 7 and stored for other purposes. For the filtered liquids, a 3M HCl solution was added until a pH of 3.5 to promote the SPP isoelectric precipitation. The protein was then washed with DI water and centrifuged for 20 minutes, at 3600 RPM, to ensure optimal separation. The SPP samples were then frozen with liquid nitrogen and lyophilized in a Labconco FreeZone 6 freeze-dryer (Missouri, United States) for 48 hours. This resulted in SPP granules that were then ground to achieve protein powder and ensure ease of dissolution later in the hydrogel manufacturing section. The obtained protein powder was stored in a cool and dry place.

2.3. Preparation of hydrogels

The same methodology was applied for the preparation of all hydrogels –squid pen protein and just chitosan (Fig. S3a). First, the SPP powder was added to a 2 wt% acetic acid solution, in a 50 mL falcon tube, with varying concentrations according to the presented DoE (Table S2). These were mixed using a SciLogex SK-O330-Pro shaker at 500 RPM for 3 hours at room temperature. Once the protein was fully dissolved, the chitosan powder was added and manually mixed in, due to the development of a highly viscous mixture, until the chitosan was fully dissolved[2]. Following this, the 1% glutaraldehyde solution was added to each hydrogel and vigorously and manually mixed in to ensure homogeneous distribution throughout the sample[3]. Subsequently, each sample was frozen using liquid nitrogen, followed by lyophilization in a Labconco FreeZone 6 freeze-dryer (Missouri, United States) for 48 hours, finally resulting in solid hydrogels with an average of 0.5wt% moisture (Fig. S3b) that became spongy upon hydration (Fig. S3c). The obtained hydrogels were stored in their respective 50 mL falcon tubes and placed in a dry and cool place.

(a)

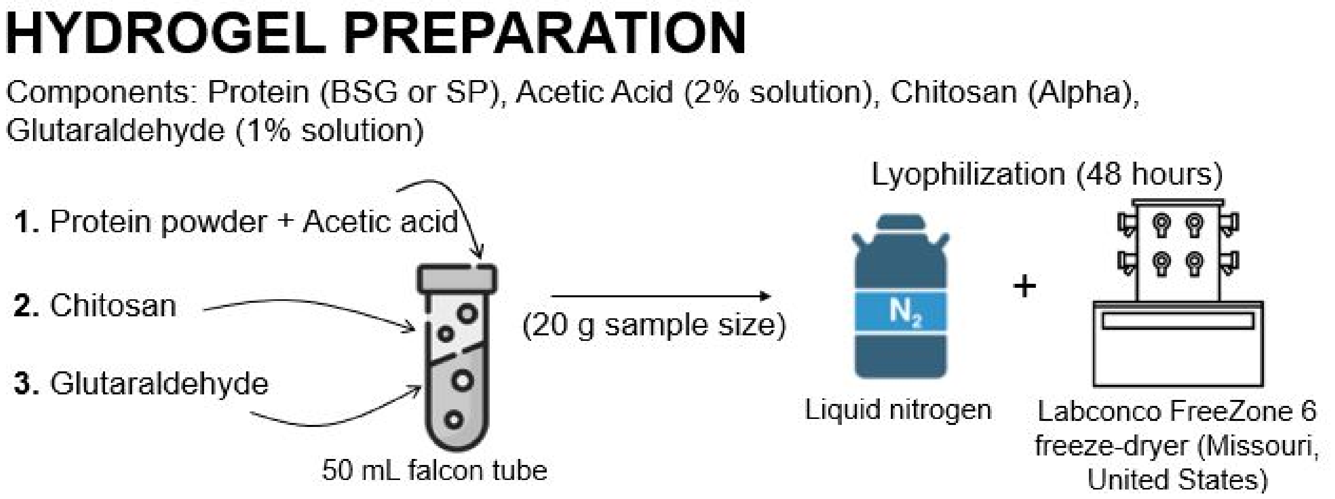

(b)

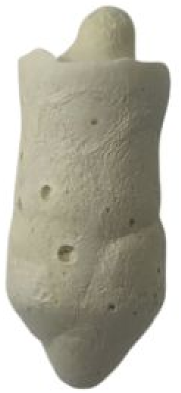

(c)

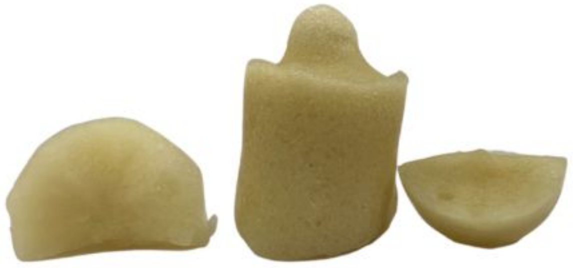

Fig. S3. (a) SPP hydrogels preparation. (b) Hydrogel after lyophilization. (c) Hydrogel after hydration.

2.4. Hydrogel Characterization

To characterize the functional groups present on each hydrogel and identify the formed bonds, Fourier transform infrared (FT-IR) spectroscopy was performed using the Agilent Cary 630 spectrometer (California, United States), with a 2 cm-1 resolution and diamond-ATR. High-resolution images of the hydrogel surfaces were obtained with the Leica DM 2500 optical microscope (Wetzlar, Germany), using objectives with magnifications and numerical apertures of 10x/0.25 and 4x/0.1, and rectangular glass slides to secure the sample.

2.5 Zeta potential

Zeta potential determination was determined with an Anton Paar Litesizer 500 (Graz, Austria) equipped with an omega cuvette. Each component to be tested was suspended in DI water at room temperature or a pH 4 buffer of Potassium Hydrogen Phtalate (Merck, Rahway, USA) . For each sample the instrument performed 250 scans (70 runs each), with an initial equilibration time of 30 seconds, at 25°C and a constant voltage of 40 mV. Values of the viscosity and refractive index were set at 0.8872 cP and 1.330, respectively. All experiments were performed in triplicate.

2.6. Water absorption capacity

Once the hydrogels were lyophilized, their water absorption capacity, also known as swelling ratio (SR) or equilibrium swelling degree (ESD), was evaluated through the following procedure. The dry hydrogel weights, Wd, were measured before fully submerging each gel in a DI water bath for 24 hours at room temperature [5]. The swelling ratio testing was conducted in separate and sealed 50 mL falcon tubes for each sample. Subsequently, the swollen hydrogels were removed from each water bath, followed by "the removal of excess surface water with a filter paper," and the equilibrium swelling weight, We, was calculated [6]. Finally, the hydrogel swelling ratio was obtained through Equation (2) [6].

$$SR = \frac{W_e - W_d}{W_d} \times 100\% \tag{2}$$

2.7. Dye adsorption capacity and removal efficiency

For both RB4 and MO equilibrium adsorption experiments, the aim was to evaluate the effect of the varying compositions and components in each hydrogel, on their dye adsorption capacity. First, dye solutions with varying concentrations between 10-200 ppm, for RB4, and 10-100 ppm, for MO, were prepared by dissolving the dye in DI water. Their corresponding absorbance, at λ_{max}, was then measured using a Shimadzu UV-2600 ultraviolet-visible spectrophotometer (Kyoto, Japan), with a wavelength range of 450-650 nm for RB4 (λ_{max}= 595) and 300-550 nm for MO (λ_{max}= 460)[7]. With the absorbance values for each concentration, it was then possible to model a linear regression and obtain a calibration curve for each dye. To evaluate the adsorption capacity of each hydrogel, a 100 ppm solution of each dye was prepared. To a 50 mL falcon tube, 20 mL of each dye solution and swollen hydrogel, with a dry weight of 0.04 g, were added. These were mixed with a SciLogex SK-O330-Pro shaker (Connecticut, United States) at 300 RPM for 24 hours. Once the dye-containing hydrogels were removed, the final concentration of each dye solution sample was measured, and the adsorption capacity and removal efficiency were calculated. Equations (3) and (4) were used to obtain the hydrogel equilibrium adsorption capacity, qe (mg/g) and removal efficiency, R%, respectively.

$$q_e = \frac{(C_0 - C_e) \times V_{dye}}{M_{dh}} \quad (3)$$

$$R\% = \frac{C_0 - C_e}{C_0} \times 100\% \quad (4)$$

C0 (mg/mL = 10-3 ppm) is the initial concentration of the dye solution, Ce (mg/mL = 10-3 ppm) is the final concentration of the dye solution, Vdye (mL) is the volume of the dye solution, and Mdh is the weight of the dry hydrogel [8 5].

2.8. Adsorption Isotherm - theory

To further study and characterize the adsorption behavior of both dyes with the hydrogels, adsorption isotherms were modelled for each relationship, specifically following the Langmuir theory. It is worth mentioning that both Langmuir and Freundlich models were tested (Fig. S3). But Langmuir's provided a better fitting for the data. The experimental data was obtained by first preparing 20 mL dye solutions with varying concentrations between 80-300 ppm, for both reactive blue and methyl orange, and placing them in 50 mL falcon tubes. To each solution, swollen hydrogels with a 0.04 g dry weight were added. These were mixed for 24 hours using a SciLogex SK-O330-Pro shaker (Connecticut, United States) at 300 RPM. The final concentration of each solution, Ce, was measured, and the equilibrium adsorption capacity, q_e, was calculated using Equation (3).

The Langmuir isotherm assumes that the adsorbent, the hydrogel, has a uniform surface, meaning that its finite adsorption sites have an equal affinity towards the adsorbate. This theory also assumes that the adsorbate molecules form a monolayer over the hydrogel's surface, meaning that only one dye molecule will fit in each adsorption site. Equation (5) describes the Langmuir isotherm. KL and αL represent the Langmuir constants, and (KL/αL) is equal to q_{max}, the theoretical maximum adsorption capacity of the adsorbent.

$$q_e = \frac{K_L C_e}{1 + \alpha_L C_e} \quad (5)$$

Equation (6) is the linearization of Equation (5), from which the Langmuir constants can be found.

$$\frac{C_e}{q_e} = \frac{1}{K_L} + \frac{\alpha_L}{K_L} C_e \quad (6)$$

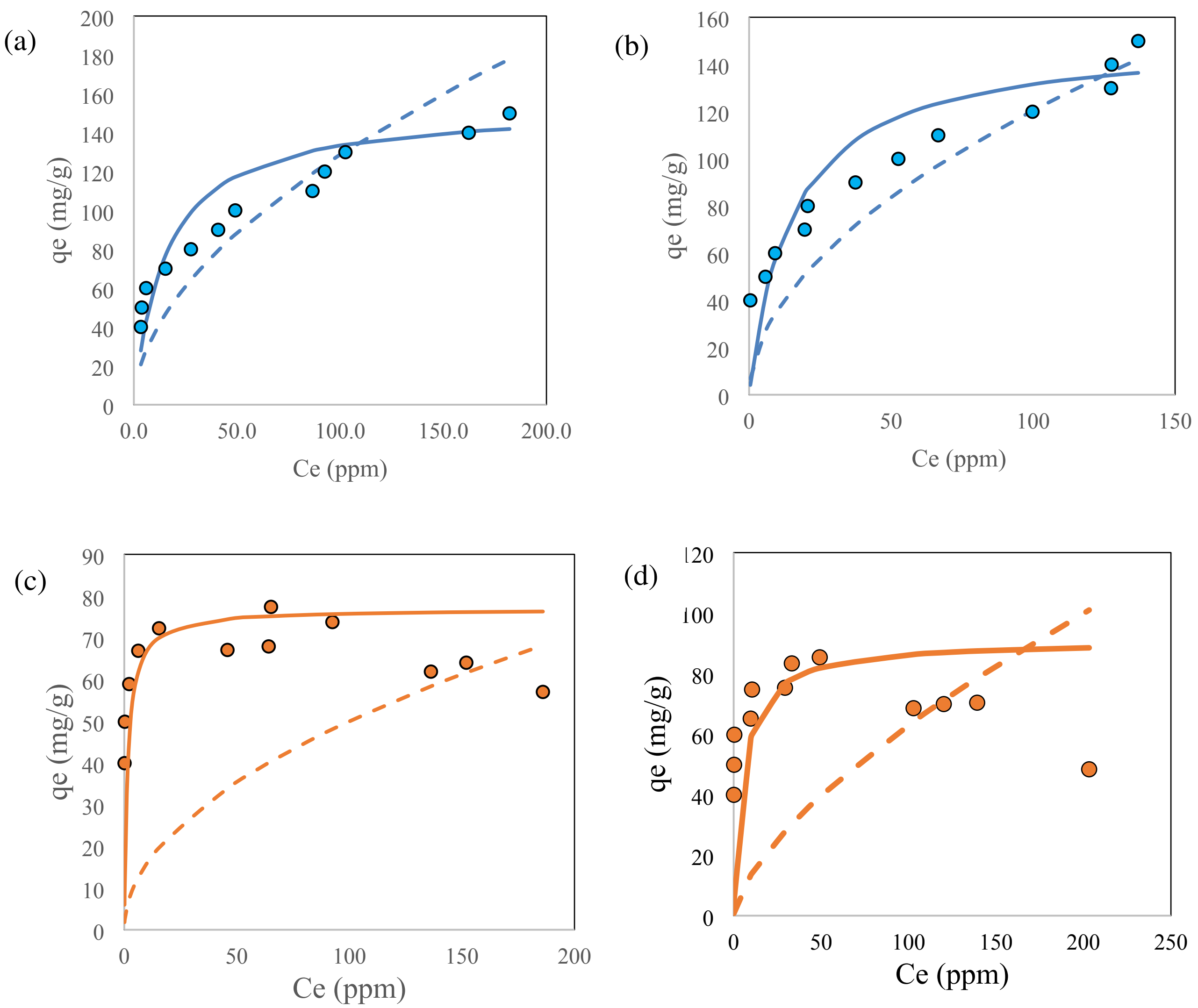

Fig. S4. Equilibrium isotherm experiments for the adsorption of RB4 with hydrogels 70_1_5 (a) and 50_1_5 (b); and adsorption of MO with hydrogels 70_1_5 (c) and 50_1_5 (d). The circles represent experimental points, the filled lines represent the Langmuir isotherms and the dashed lines represent the Freundlich isotherms.

With the experimentally found values of C_e and q_e, C_e/q_e was plotted against Ce. The y-intercept of the linear plot is equal to (1/KL), from which KL was found, and the slope is equal to (αL/KL), from which αL was found. Lastly, the graphical representation of the Langmuir isotherm was found by applying the constants and experimental Ce values to Equation (5). The characteristic plateau of this theory demonstrates that once the adsorption capacity reaches qmax, the adsorbent surface is saturated and no further adsorption takes place [9].

2.9 Adsorption parameters study – RB4

2.9.1 Temperature and time (kinetics)

To evaluate the impact of temperature and time, three temperatures, 25°C, 45°C and 65°C were studied. Hydrogel 50_1_5 was chosen due to its performance in the equilibrium DoE experiments. The experiment was performed in triplicates with 0.2 wt% hydrogel loading for 24 h, pH = 7 and 300 RPM stirring speed. Aliquots of 1 mL were taken at 0, 2, 3, 4, 6, 12 and 24h. The samples were then immediately analyzed via UV-vis at λ = 595 nm.

2.9.2 Effect of pH

Once the optimum temperature and time (65°C and 6h) were chosen, different pHs were evaluated. A sodium hydrogen phthalate pH = 4 buffer (Merck, USA) was adjusted to pH = 1 and 2 with a 3M HCl solution. A di-sodium hydrogen phosphate buffer pH = 7 was adjusted to pH = 6 with a 3M HCl solution and to pH = 8 with a 3M NaOH solution. The experiment was performed in triplicates with 0.2 wt% hydrogel loading for 6 h and 300 RPM stirring speed.

2.9.3 Effect of adsorbate dose

The effect of RB4 concentration was studied with different initial concentrations of the dye, 30, 60, 100, 150 and 200 ppm were used. The experiment was performed in triplicates with 0.2 wt% hydrogel loading for 6 h, pH = 7 and 300 RPM stirring speed.

2.9.4 Kinetics of adsorption - theory

The adsorption rate is proportional to the first power of concentration in the pseudo-first-order kinetic model. Equation 7 displays the linearised model equation (Wong et al. 2020).

$$ln(q_e - q_t) = \ln q_e - \frac{k_1 t}{2.303} \qquad (7)$$

where t is the contact time (min), k_1 is the pseudo-first-order kinetic model's rate constant (min^{-1}), and qt is the adsorption capacity at instant time (mg/g). To find k_1 and predicted/theoretical qe (from slope and intercept), respectively, ln (q_e–q_t) was plotted against t using the linearised equation of the pseudo-first order kinetic model. Conversely, the pseudo-second-order kinetic model is associated with the function of chemisorption as the adsorption process's rate-controlling phase. Equation 8 shows the linearised equation.

$$\frac{1}{q_t} = \frac{1}{k_2 q_e^2} - \frac{1}{q_e} \qquad (8)$$

where k_2 is the rate constant of pseudo-second-order kinetic model (g mg^{-1} min^{-1}). From the linearized equation of pseudo-second order kinetic model, a graph of t/qt is plotted against t to determine k_2 and predicted/theoretical q_e (from slope and intercept) respectively.

2.10 Desorption experiments – RB4

Several different types of solvents were tested to remove RB4 from hydrogel 50_1_5 after the adsorption. The solvents were ethanol, DI water, NaOH (0.5 and 1 M), acetone and [Ch][OAc]. The experiment was performed in triplicates with 0.2 wt% hydrogel loading for 6 h, at 65°C, pH = 7 and 300 RPM stirring speed. After the experiment, the hydrogels were inspected visually and via an optical microscope Leica DM 2500 optical microscope (Wetzlar, Germany),

2.11 XPS

X-ray photoelectron spectra were recorded on a Thermo Fisher K-Alpha Photoelectron Spectrometer equipped with a 180° double focussing hemispherical analyser, 128-channel detector, and monochromated Al Kα microfocused x-ray source (hν = 1486.6 eV) operated at 6 mA emission current and 12 kV anode bias. Prior to XPS measurements, samples were degassed on a Schlenk line at <1 x 10-2 mbar for 24 h before mounting 5-10 mg on a welled copper plate (Nakasu et al. 2020).

After degassing to <3 x 10-7 mbar in the sample transfer chamber, samples were introduced to the analysis chamber, which operates with a base pressure of <2 x 10-9 mbar. A spot size of 400 µm and pass energies of 200 eV (survey) and 20 eV (high resolution) were used during analysis.

Typically, 60 scans with a dwell time of 50 ms were used for HR scans; hence each HR scan equals ≈ 10 m of X-ray exposure. Samples were prevented from charging with a dual-beam flood source. Survey scans were quantified in Avantage 5.951 using smart backgrounds and ALTHERMO1 RSFs. High resolution scans were converted to .VAMAS files and processed in CasaXPS 2.3.19. Scans were charge referenced to the C 1s aliphatic signal (component 1) at 285.0 eV and each photoemission was fitted with the minimum number of GL (30) lineshapes (with spline-linear backgrounds) required to replicate the photoemission signals. C 1s components had FWHM constraints (1-1.3 eV), however, O 1s components were unconstrained. C 1s and O 1s.

3. Results and discussion

3.1 Zeta potential

To further understand the difference in the hydrogels reactivity toward the dyes, the zeta potential (ζ) of both dyes was investigated. Additionally, the zeta potential of chitosan and SPP were also analysed and compared to understand the positive contribution provided by the squid pen protein to the adsorbents.

Zeta potential compares the difference between the surface potential of a particle to the potential of the liquid it is dispersed in. Therefore, this representation of surface change is useful when investigating how the dispersed particles interact with other surfaces (Dai 1994)

The zeta potential values obtained were the following: for chitosan ζ= +14.85 mV, for squid pen protein ζ= +26.64 mV, for RB4 ζ= -16.32 mV, and for MO ζ= -39.79 mV. The point of zero charge of the squid pen protein was observed at pH 3.5.

Chitosan is a polycation, with the amino groups which can be easily protonated. Hence, it presents positive zeta potential values when dispersed that facilitate the adsorption of the dyes, which are negatively charged. In a study by Athavale et al. (2022) (Athavale et al. 2022), the zeta potential values of chitosan nanoparticles produced were e between 12-25 mV, similar values to the values s obtained in this work.

The fact that SPP had a positive zeta potential in DI water means that the protein likely contained residual hydrochloric acid from the isoelectric precipitation. Protein isolates normally present positive zeta potential at low pH (2-4) and negative values at high pH (above 6) (Freitas et al. 2017). Notably, the magnitude of SPP zeta potential was higher than the chitosan. The increase in surface charge resulted in stronger interactions between the hydrogel and dyes, and therefore higher adsorption capacity and dye removal efficiency results (Athavale et al. 2022).

Both anionic dyes presented negative zeta potential values, which partly explains the efficacy of the biocomposites hydrogels and the dyes in terms of electrostatic interactions. Both dye molecules present sulphonic acid groups and therefore, their pKa is below 7. MO's pKa is 3.42, and RB4's first pKa is 0.8 (Bagchi and Ray 2015)), which means that in DI water these molecules should be negatively charged. MO, however, presented relatively higher magnitude of zeta potential values, which suggest greater removal efficiency. Regarding such apparent discrepancy, it is also worth noting that while intermolecular and covalent bonds that can also play a role in the adsorption. RB4, as previously stated, can covalently bind to the adsorbent, which may explain the higher adsorption values.

3.2 Study of parameters that influence adsorption of RB4 to the hydrogel 50_1_5

3.2.1 Temperature

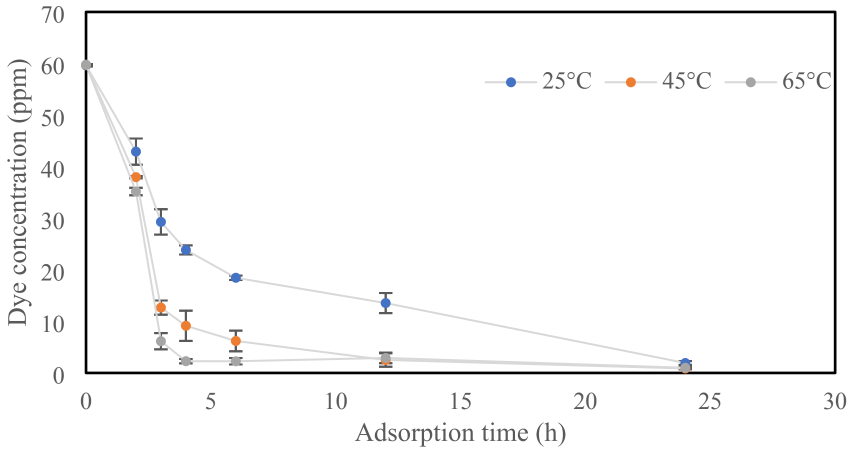

Figure S5. Effect of temperature on the RB4 adsorption performed over 24 h , with an initial dye concentration of 60 ppm and adsorbent concentration of 0.07g per 20 mL of dye solution. The standard errors were obtained from triplicates.

3.2.2 Kinetics of adsorption

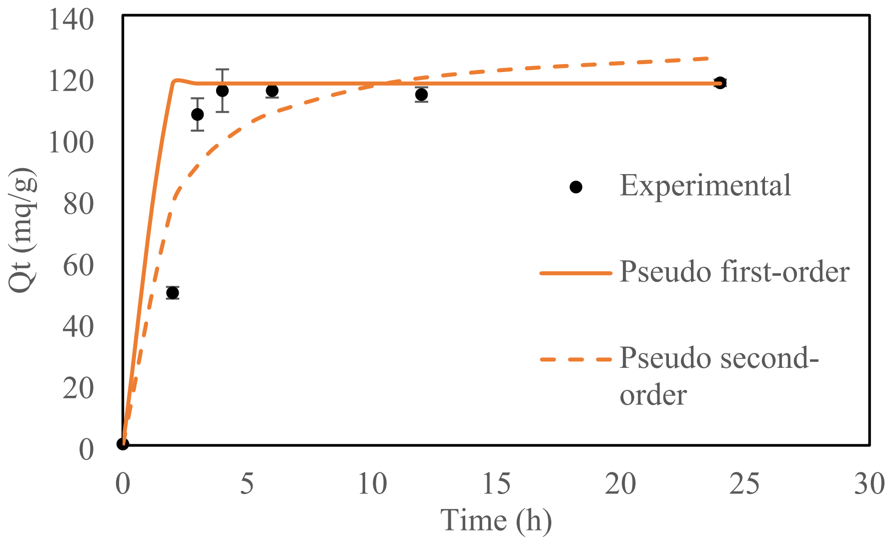

Figure S6. Comparison of two adsorption kinetic models, pseudo first order and second order with the experimental data points of RB4 adsorption performed with hydrogel 50_5_1 at 65 °C over 24 h , with an initial dye concentration of 60 ppm and adsorbent concentration of 0.07g per 20 mL of dye solution. The standard errors were obtained from triplicates.

3.2.3 Effect of pH

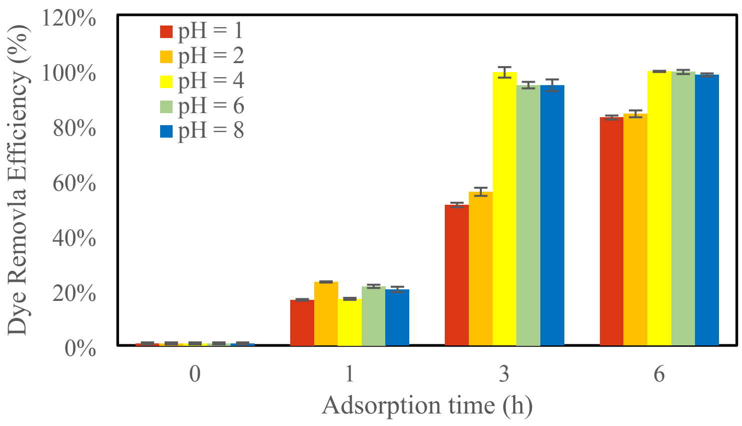

Figure S7. Effect of using different pH's on the adsorption of RB4 to hydrogel 50_5_1 at 65 °C over 24 h , with an initial dye concentration of 60 ppm and adsorbent concentration of 0.07g per 20 mL of dye solution. The standard errors were obtained from triplicates.

3.2.4 Effect of adsorbate dose

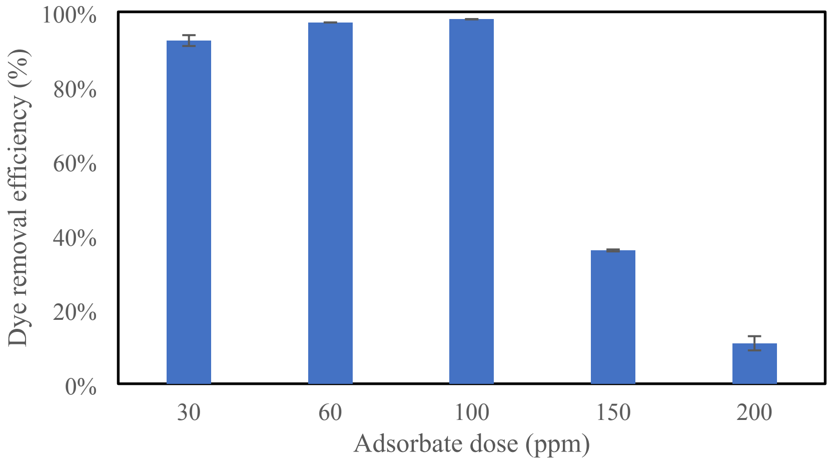

Figure S8. Effect of increasing initial concentration of RB4 on the performance of hydrogel 50_5_1 at 65 °C over 24 h , with an initial dye concentration of 60 ppm and adsorbent concentration of 0.07g per 20 mL of dye solution. The standard errors were obtained from triplicates

3.2 FT-IR

Table S11. FT-IR absorption bands with corresponding chemical groups [10]

Wavenumber (cm^{-1})	Chemical group
3251	Stretching vibrations of O-H
2866	Stretching vibration of C-H in alkanes
2102	Stretching vibration of C≡N
1635	Bending vibration of N-H amide I
1521	Bending vibration of N-H amide II
1399	Asymmetric deformation of CH_3
1375	Symmetric deformation of CH_3
1248	Stretching vibration of C-N amine III
1151	Stretching vibration of C-O
1062	Stretching vibration of C-O in chitosan/ether

1017	Stretching vibration of C-O in polysaccharides/ester
889	bending vibration of C-H in alkanes

3.3 XPS analysis

Table S12. Low resolution elemental survey

	C	O	N	Si	S	Cl	Na	Ca
RB4	57.8	20.0	9.0	0.6	3.2	4.5	3.7	0.3
Hydrogel before adsorption	73.0	18.8	5.0	0.5	0.5	0.4		1.6
Hydrogel after adsorption	62.4	22.7	8.2	2.0	1.5	0.8	0.7	0.4

ABBREVIATIONS

GLA – Glutaraldehyde. MO – Methyl orange. SPP- Squid pen protein. RB4 -Reactive blue 4.

REFERENCES

Allen, S. J., Mckay, G., & Porter, J. F. (2004). Adsorption isotherm models for basic dye adsorption by peat in single and binary component systems. Journal of Colloid and Interface Science, 280(2), 322–333. https://doi.org/10.1016/j.jcis.2004.08.078

Fan, J., Shi, Z., Lian, M., Li, H., & Yin, J. (2013). Mechanically strong graphene oxide/sodium alginate/polyacrylamide nanocomposite hydrogel with improved dye adsorption capacity. Journal of Materials Chemistry A, 1(25), 7433. https://doi.org/10.1039/c3ta10639j

Gao, H., Jiang, J., Huang, Y., Wang, H., Sun, J., Jin, Z., Wang, J., & Zhang, J. (2022). Synthesis of hydrogels for adsorption of anionic and cationic dyes in water: ionic liquid as a crosslinking agent. SN Applied Sciences, 4(4). https://doi.org/10.1007/s42452-022-04996-z

Interpretation of Infrared Spectra . (2023, July 11). Stanislaus State .

IR Spectrum Table & Chart . (2023). Merck.

JMP. (2023). Design of Expriments. JMP Statistical Discovery .

Lu, Z., Zou, L., Zhou, X., Huang, D., & Zhang, Y. (2022). High strength chitosan hydrogels prepared from NaOH/urea aqueous solutions: the role of thermal gelling. Carbohydrate Polymers, 297, 120054. https://doi.org/10.1016/j.carbpol.2022.120054

M. Bower, K. (2023). What Is Design of Experiments (DOE)? American Society for Quality .

Mirzaei B., E., Ramazani S. A., A., Shafiee, M., & Danaei, M. (2013). Studies on Glutaraldehyde Crosslinked Chitosan Hydrogel Properties for Drug Delivery Systems. International Journal of Polymeric Materials, 62(11), 605–611. https://doi.org/10.1080/00914037.2013.769165

Smith, B. (1998). Infrared Spectral Interpretation - A Systematic Approach . CRC Press. https://doi.org/10.1201/9780203750841

Tsai, F.-C., Ma, N., Chiang, T.-C., Tsai, L.-C., Shi, J.-J., Xia, Y., Jiang, T., Su, S.-K., & Chuang, F.-S. (2014). Adsorptive removal of methyl orange from aqueous solution with crosslinking chitosan microspheres. Journal of Water Process Engineering, 1, 2–7. https://doi.org/10.1016/j.jwpe.2014.02.001

Wang, S., Chen, X., Yin, Y., Meng, H., Wang, Y., Xiao, Z., Wang, H., Liang, D., & Xie, Y. (2022). Lignin-based hydrogels for efficient dye removal via synergistic effect of multiple interactions. Industrial Crops and Products, 189, 115840. https://doi.org/10.1016/j.indcrop.2022.115840
